# The effect of internal marketing on job satisfaction in health services: a pilot study in public hospitals in Northern Greece

**DOI:** 10.1186/1472-6963-11-261

**Published:** 2011-10-09

**Authors:** Efthymios Iliopoulos, Constantinos-Vasilios Priporas

**Affiliations:** 1 Orthopaedic Department, General Hospital of Veria, 59100 Veria, Greece; 2 University of Macedonia, Department of Marketing and Operations Management, 58200 Edessa, Greece

## Abstract

**Background:**

The purpose of this study was to explore the effect of internal marketing on job satisfaction in health services, particularly in public hospitals in Northern Greece.

**Methods:**

A questionnaire with three sections was used. The first one referred to internal marketing by using Foreman and Money's scale, while the second one contained questions on job satisfaction based on Stamps and Piermonte's work. The last section included demographic questions. Three categories of health care professionals, nurses, doctors and paramedic personnel working in public hospitals have participated.

**Results:**

Doctors tend to be more satisfied with their job than nurses in the same hospitals. Male personnel also tend to be more satisfied with their job than female. Time-defined work contract personnel have a greater level of job satisfaction than permanent personnel. Marital status, position, and educational level have no statistically significant impact on job satisfaction. A slight decline in job satisfaction occurs as the personnel age.

**Conclusions:**

Internal marketing has a positive effect on the job satisfaction of hospital staff in Northern Greece. Also, doctors and male personnel seem to have greater levels of job satisfaction. Staff with time-defined work contracts with the hospital are more satisfied than permanent staff, and as the staff age, there is a slight decline in job satisfaction.

## Background

Internal marketing refers to all the actions that an organization (i.e., health care organizations, hospitals) has to perform in order to develop, train and motivate its employees, so to enhance the quality of the services provided to its customers [1]. Internal marketing also enhances the productivity of the employees and improves customers' satisfaction which increases earnings [2]. This is important even for the public non-profit healthcare system in Greece, because increased customers' satisfaction means the hospital will treat a greater number of patients, which will increase the hospital funds from the insurance companies. Kotler stated that internal marketing is the task of successfully recruiting, educating and motivating employees so as to perfect customer service [3]. He also mentioned that it is not logical to expect perfect services from an organization, whose employees are not ready to provide such services. Internal marketing is the way to accomplish that [4]. Other authors correlate internal marketing with human resources and change management [5]. Some authors suggest that successful organizations are those that develop evenly internal and external marketing [6]. Papasolomou [7] points out that the objective of internal marketing is to create motivated and customer-conscious employees in order to achieve service excellence.

Administering their human resources effectively is very important for organizations that provide services to their customers, because the services generated by the organization are produced by the employees directly for the client [1]. This necessity led to the development of internal marketing as a distinct field of marketing.

In 1970s Berry and his colleagues [8] were the first to introduce the concept of internal marketing in the US, based on the traditional marketing mix of 4 Ps (Product, Price, Promotion and Place). They posited that employees can be considered to be internal customers and their jobs could be seen as the organization's products. So, the organization tries to treat employees in ways which enforce job satisfaction and motivate them to be more productive [9]. This approach mainly focused on satisfying and motivating employees. The general idea is that in order to have satisfied customers, a company has to have satisfied employees at first. So by meeting employees' needs, the company manages to make them more capable of satisfying the customers, which is very important especially for organizations which provide services [10]. Berry and Parasuraman mention that because of internal marketing, certain conditions are created in the organization, which encourage and inspire the employees in their work [11].

In the 1980s, Gronroos [12,13] set the basis for the Scandinavian stream of thought on internal marketing. He argued that employees were an important part of the overall product or service delivered. Therefore, they should be trained as marketers with customer retention skills that would enable the building of ongoing customer relationships. This approach centered on creating customer oriented behavior. Gronroos stated that internal marketing has to create the kind of internal environment in the organization which will lead the employees to satisfy customers. In addition, Gronroos et al mention that good communication between employers and employees is very important for the company [12]. Conduit and Mavondo, based on Gronroos basic idea, divided internal marketing practices into five categories: employees' education, management support, internal communication, human resources and employees' intervention in external communication [14].

In the 1990s, Rafiq and Ahmed [15,16] in the UK developed a hybrid approach to internal marketing based on the two previous approaches. They stressed that internal marketing was inherently difficult to implement because of inter-functional conflicts between departments, management and employees, and the firm's inherent resistance to change. This approach is considered to be the basis for the development of cross-functional integration within the firm. The same authors in 2000 [2] identified the main elements of internal marketing. From the analysis of the key literature these are: employee motivation and satisfaction, customer orientation and customer satisfaction, inter-functional co-ordination and integration, marketing-like approach to the above, implementation of specific corporate or functional strategies. Based on these, they define IM as "*a planned effort using a marketing-like approach to overcome organizational resistance to change and to align, motivate and interfunctionally co-ordinate and integrate employees towards the effective implementation of corporate and functional strategies in order to deliver customer satisfaction through a process of creating motivated and customer-orientated employees" *[2].

Employees as internal customers have to be satisfied with their job first before they can satisfy the customers of the organization [9,17]. Foreman and Money [17] developed a questionnaire with 15 questions in order to measure internal marketing in an organization. The questions of this questionnaire are divided into three groups: How the employees understand the vision of the organization (Vision), their personal development in the organization (Development) and the payroll system (Rewards).

In conclusion we can state that internal marketing as a concept or management practice contributes to higher quality services. Ahmed and Rafiq [15] stress that though the need for internal marketing is understood, the reality unfortunately demonstrates that only a few organizations implement internal marketing in practice. This is largely due to the lack of an agreed definition of internal marketing by researchers [2], and due to the fact that many service organizations consider it to be a vague concept, and thus they fail to understand the importance and meaning of internal marketing and to address organizational level issues and challenges [18].

### Job Satisfaction

The importance of job (work) satisfaction in services marketing literature is not new. As a theoretical notion, it was first presented by Hoppock in the mid 1930s [19]. He indicated that an employee's job satisfaction is derived from the mental and physical satisfaction they experience in the work environment and from the work itself. Job satisfaction in general refers to the emotions the employee feels about his job, and how he reacts to them [20,21]. Job satisfaction is a widely studied concept in many different occupational areas, including the health care industry. Nelson [22] contends that the principal determinant of whether health care employees stay or voluntarily quit a job is dissatisfaction with their employment situation.

Job satisfaction has been defined as an attitudinal reflection of the extent to which people like or dislike their jobs [23]. It also shows how an employee feels about his job [24]. For other authors job satisfaction refers to a joyful or positive emotional state regarding work or work experience [25,26]. However, the definition of job satisfaction differs according to where emphasis is put by each scholar. Bussing et al [27] contend that job satisfaction is based on the desires, needs, motives, and feeling in the working environment, i.e., the satisfaction or dissatisfaction of an employee with his/her work. Robbins sees job satisfaction in relation to the employee's behavior. The higher the job satisfaction is the better the employee's behavior is [28].

The grade of job satisfaction depends on the difference between the prospective gains from the job and the actual gain from it [29]. A study among caregivers in the USA concluded that employees who perceived the quality of care to be high, had higher levels of job satisfaction [30]. Also, it was found that there is a positive correlation between nurses' job satisfaction and the quality of health services [31,32]. On the other hand, job stress amongst nurses seems to have a negative effect on job satisfaction. Job satisfaction is not a unitary concept, but rather the opposite. A person can be relatively satisfied with one aspect of his or her job but dissatisfied with other aspects [33]. Much of the ongoing interest in job satisfaction is based on the belief that job satisfaction is highly related to employee loyalty, job performance and retention [34].

Porter and Lawler [35] separate job satisfaction into internal and external satisfaction. Internal satisfaction consists of all the factors that have direct correlation with job satisfaction (i.e., the sense of success, independence, job rotation, job opportunities, personal development, creativity, self respect, etc). External satisfaction consists of all the other factors which indirectly correlate with job satisfaction (i.e., job environment, interpersonal relations between colleagues, high salary and possibility of promotion).

In general, it could be said that satisfied employees produce better services, because there is a positive correlation between job satisfaction and customer satisfaction [36]. This is true especially in the services industries, where the intangible nature of the services makes employees one of the most crucial parameters in the value generation process of the service organization.

Smith et al, in 1969, developed the Job Description Index (JDI) in order to measure job satisfaction. This index consists of fields such as the type of job, supervision and colleagues' relations [37]. Spector, in 1985, upgraded that index by developing the Job Satisfaction Survey (JSS). This survey studies more parts of job satisfaction, such as motivation, employees' prosperity and wages [23]. Stamps and Piedmont, in 1986, adapted JSS to health services, and created a new questionnaire [38].

### Relationship between internal marketing and job satisfaction

The fundamental concept of internal marketing is to treat employees at all levels of the organization as internal customers [12,39]. The growing acknowledgment of the importance of the employees' role has led service organizations to adopt internal marketing and hence, treat their employees as internal customers [40]. Previous studies showed that internal marketing has a positive effect on job satisfaction [2,14,41-43].

Studies in health services found that customers' satisfaction was greater in organizations where employees were sensitized by internal marketing to produce high quality services to the customers. Chang and Chang in their study in two hospitals in Southern Taiwan proved that internal marketing had a positive influence to nurses' job satisfaction [44]. Peltire et al [45] in their study in a health care organization in the USA, in the internal marketing context, found that overall the participant employees (nurses) were satisfied with their job while their perceptions of the quality of care had the greatest impact on explaining differences in employees' level of job satisfaction. Other studies depicted that internal marketing could reduce turnover intention in medical personnel at dangerous illness outbreaks [1].

### Aims of the study

Few of the internal marketing studies have centred on health care services. Chang and Chang [44,46] and Peltire et al [45] in their studies in hospitals focused on nurses, while Cooper and Cronin [47] in their study in a nursing home care focused on nursing assistants. Taking into consideration the limited number of research studies in the field, the aim of this study is to explore the effect of internal marketing on job satisfaction in health services and particularly in public hospitals in Northern Greece. Three categories of health care professionals, nurses, doctors and paramedic personnel were used for the purposes of the study.

### Research hypothesis

As described above, both internal marketing and job satisfaction contribute to higher quality of services. It is interesting to explore this case by focusing in internal marketing that takes place in hospitals in Greece, in order to increase the employees' job satisfaction, which will consequently contribute to higher quality of health services in our health care system. Therefore, our hypothesis is that: *Internal marketing in Greek hospitals has a positive effect on employees' job satisfaction*.

## Methods

### Sample and data collection

The study was conducted in three hospitals of Northern Greece. Doctors, nurses and paramedics were included in the study. These professions are the main branch of a hospital's personnel which in fact produce the health care services of a hospital. Because of this, we focused on these types of personnel. The study was approved from the head manager of each of the three hospitals, because there is no ethics board in public hospitals in Greece.

All questionnaires were collected in May 2010 and they were completed anonymously. Researchers were nearby while the questionnaires were completed, in order to avoid any potential bias or influence from the researchers and also to be able to provide any assistance, if necessary. This practice helped us to have a very high response rate. We finally collected 450 questionnaires out of a total of 482 by using a convenience sample. Response rate was 93.3% (some employees were too busy to complete the questionnaire or stopped the procedure before finishing). Also problems of misunderstanding were dealt with immediately. All participants gave their informed consent prior to the study and the questionnaires were anonymous, so there were no ethical problems for the participants.

### Questionnaire

In order to conduct our study we designed a questionnaire, which contained three groups of questions. Each questionnaire came with a small letter from the researchers, where the purpose and the confidentiality of the study were described. The first group of questions refers to internal marketing. Money and Foremore's questionnaire was translated and used, in order to help us measure the internal marketing of these hospitals [17]. Participants could answer the fifteen questions of the questionnaire by using a five point Likert scale, where the score of agreement or disagreement is stated [48]. Job satisfaction was investigated with the second group of questions where questions from Stamps and Piermonte's questionnaire were used [38]. Although the original instrument has two essential parts, only one part was used in the present study so as to facilitate ease of use [49]. A five point Likert scale was used for this section too. Both scales were checked for content validity through expert review and reliability with a Cronbach's alpha of 0.91 and 0.74 respectively, which means that both scales were internally reliable. The third group, comprised of ten questions, helped us to form the demographic and occupational profile of the sample. Our questionnaire can be cited in Additional file 1.

### Research variables and analysis

The variables of the study were internal marketing and job satisfaction. Internal marketing was the independent variable and job satisfaction was the dependent variable. In order to analyze the results we had to create scores for these two variables. All questions used a 5-point scale ranging from 1 = totally disagree to 5 = totally agree. By adding the points from each question, a total score for internal marketing was created for each questionnaire. Theoretically this score could vary from 15 to 75. A similar score system was also developed for job satisfaction. The five point Likert scale, which was used to measure internal marketing, was also used to measure job satisfaction. The negative questions were reversed, so the score could reflect more accurately the personnel's job satisfaction. The higher the score was the higher the job satisfaction was. Theoretically the score for job satisfaction could vary from 14 to 70.

The data analysis was performed with SPSS version 15.0. The hypothesis was tested with Spearman ρ test (Linear Bivariate Correlation). Mann-Whitney U test was used in order to check job satisfaction with demographics [50]. Nonparametric tests were used because the job satisfaction scores were not normally distributed (Kolmogorov-Smirnov test = 0.016)

## Results

There were 450 questionnaires collected out of 482 in total. The response rate was 93.3% (some employees were too busy to complete the questionnaire or stopped the procedure before finishing). Our presence when the questionnaires were being completed helped us to have this high response rate. Of the personnel who participated in the survey 66.2% were female (298 questionnaires), and 33.8% were male (152 questionnaires). Nurses made up 50.2% (n = 226), doctors 36.9% (n = 166) and paramedic personnel 12.9% (n = 58) of the total sample. The age of the personnel who answered the questionnaire varied from 18 to 66 years old, with an average of 38.77 years. Three hundred and two (302) employees had a permanent contract with the hospital (67.1%) and 148 (32.9%) had a time-defined contract with the hospital. Three hundred (300) were married (66.7%) and 150 (33.3%) were single. Job experience varied from 1 to 35 years with men having 12.95 years experience on average. The demographic profile of the participants can be seen in detail on Table 1.

**Table 1:** Demographic profile of respondents

	Doctors		Nurses		Paramedics		Total
***Gender***	**N**	**%**	**N**	**%**	**N**	**%**	**N**
Male	108	65.1	36	15.9	8	13.8	152
Female	58	34.9	190	84.1	50	86.2	298
TOTAL	166	100.0	226	100.0	58	100.0	450
							
***Age (years)***	**N**		**N**		**N**		**N**
Mean	36.87		40.02		39.34		38.77
Min - Max	26 - 66		18 - 57		24 - 54		18 - 66
							
***Marital status***	**N**	**%**	**N**	**%**	**N**	**%**	**N**
Married	82	49.4	178	78.8	40	69.0	300
Unmarried	84	50.6	48	21.2	18	31.0	150
TOTAL	166	100.0	226	100	58	100.0	450
							
***Contract type***	**N**	**%**	**N**	**%**	**N**	**%**	**N**
Permanent	52	31.3	208	92.0	42	72.4	302
Time-defined	114	68.7	18	8.0	16	27.6	148
TOTAL	166	100.0	226	100.0	58	100	450
							
***Job position***	**N**	**%**	**N**	**%**	**N**	**%**	**N**
Director	52	31.3	134	59.3	28	48.3	214
Subordinate	114	68.7	92	40.7	30	51.7	236
TOTAL	166	100.0	226	100.0	58	100.0	450
							
***Job experience (years)***	**N**		**N**		**N**		**N**
Mean	8.31		16.15		13.76		12.95
Min - Max	1 - 25		1 - 33		1 - 32		1 - 35
							
***Education***	**N**	**%**	**N**	**%**	**N**	**%**	
PHD	26	15.7	1	0.45	0	0	27
MSc	20	12.0	1	0.45	0	0	21
Diploma	120	72.3	4	1.8	2	3.4	126
Bachelor	0	0.0	126	55.8	32	55.2	158
Vocational Graduates	0	0.0	94	41.6	24	41.4	118
TOTAL	166	100.0	226	100.0	58	100.0	450

As mentioned above, we have developed scores for internal marketing and job satisfaction with the answers of corresponding questions for each questionnaire. The internal marketing scores varied from 15 to 74 with a mean of 33.7. Scores for job satisfaction varied from 14 to 51 with a mean of 35.98. We entered the data to SPSS and in order to check our hypothesis we used Spearman *ρ *test (Linear Bivariate Correlation). All statistics were checked at statistical significant level of 0.05. A significant correlation (p = 0.554) was found between internal marketing score and job satisfaction score. So our hypothesis was confirmed. Internal marketing has a strong statistically significant positive correlation with job satisfaction on personnel in Greek public hospitals.

In order to check the correlation of other variants with the personnel's job satisfaction, we used Mann-Whitney U test, at 0.05 level of statistical significance. A statistically significant difference was found between male and female personnel. Male personnel tend to be more satisfied with their job (p = 0.005) than female with the same job in Greek hospitals. Also an interesting finding was discovered by checking job satisfaction between jobs. Doctors tend to be more satisfied with their job (p < 0.0005) than nurses. Personnel with a time-defined work contract with the hospital were more satisfied (p = 0.001) than personnel with a permanent work contract. There were no statistically significant difference in marriage (p = 0.155) and job position (p = 0.116). Levels of education seem to have no effect on job satisfaction between nurses. No significant difference in job satisfaction was found between nurses with bachelor degrees and vocational graduates (p = 0.95). By using the Spearman test we found a weak negative correlation between job satisfaction and age (p = -0.169), and between job satisfaction and job experience (p = -0.237). All results are summarized in Table 2.

**Table 2:** Summary of Results

		Job Satisfaction		
	**Spearman *ρ *test**	**Mann-Whitney U test**	**Median**	**Scores**

**Internal Marketing**	0.554	-		
**Age**	-0.169	-		
**Job Experience**	-0.237	-		
**Gender (male/female)**	-	0.005	**Male**	**Female**
			39	35
**Specialty (doctors/nurses)**	-	< 0.0005	**Doctors**	**Nurses**
			39	35
**Contract Type (permanent/time defined)**	-	0.001	**Permanent**	**Time Defined**
			35	39
**Marital Status (married/unmarried)**	-	0.155	**Married**	**Unmarried**
			36	38
**Job Position (director/subordinate)**	-	0.116	**Director**	**Subordinate**
			36	38
**Nurses education (bachelor/vocational graduates)**	-	0.95	**Bachelor**	**Vocational Graduates**
			35,5	35

## Discussion

Our basic hypothesis was confirmed. Internal marketing that exists in the sample hospitals has a significant positive effect on personnel's job satisfaction. Similar findings were produced by Chang and Chang's study, which was focused on nurses in two medical centers in Southern Taiwan [44].

Our results about job satisfaction are interesting. We found that doctors tend to be more satisfied with their job in contrast to the nurses in the same hospitals. It seems that the nature of a doctor's job helps them to be more satisfied. Breslau et al concluded that there was no significant difference in job satisfaction between doctors and paramedic personnel. They only found a slight difference in terms of doctors being more satisfied with their salary [51].

Our results in terms of gender and job satisfaction contrast with the literature. Sibbald et al found that male general practitioners were less satisfied with their job than female ones [52]. Our findings showed that males were significantly more satisfied with their job than females. Our results could have been affected by our sample. More doctors were male than female and more nurses were female than male, so considering our previous result about job and job satisfaction, the male doctors could have affected the statistical significance of our result.

Economic crisis and high levels of unemployment in our country can explain our results about staff work contracts. Personnel with defined-time work contracts with the hospital, concerned about unemployment and insecurity, have fewer demands from their job than those who have a permanent work contract. So they are more satisfied with their job.

A weak negative correlation was found between age and job experience and job satisfaction. This can be explained by the fact that as a person ages and gets more experience, his needs and demands grow, so do the requests from his job. This can explain why as we grow older our satisfaction with our job declines.

## Limitations

The results of this study should be interpreted with several limitations in mind. Firstly, there is only bivariate statistical analysis of the results. Multivariable approaches could have been used in order to decipher the correlations of job satisfaction with internal marketing, demographic and occupational factors. Secondly, a convenience sampling method in the three sample hospitals was selected and although the sample size was adequate for the purposes of this study and the response rate was high (93,3%), it cannot be considered representative of all health care professionals working in public hospitals in Greece. Further studies should expand the research in more public hospitals from all the parts of the country, in order to have a better scope of job satisfaction and internal marketing in public hospitals in Greece. Thirdly, the measurements associated with job satisfaction are limited by the size of the questionnaire (only one part of Stamps and Piermonte's questionnaire were used) because it was designed in order to avoid respondent fatigue. Finally, emotional and other personal factors of the responders could have influenced the answers which were given in our questionnaire.

## Conclusions

The results show that our basic hypothesis was confirmed. It seems that internal marketing has a significant positive effect on job satisfaction of healthcare personnel in public hospitals in Northern Greece. Also doctors and male personnel seem to have greater levels of job satisfaction. Personnel with time-defined work contracts with the hospital are more satisfied and as the personnel age, a slight decline in job satisfaction is noted.

Hospital administrators as well as the Greek Ministry of Health should recognize that internal marketing in the healthcare service industry could be very helpful by improving the job satisfaction of healthcare employees. It will be beneficial to adopt more human resources practices in the internal marketing context. This is a high labor intensity service, involving very frequent interaction with customers-patients, since their life and health are often in danger. Therefore, it is a necessity that the personnel (doctors, nurses, paramedics) are satisfied so that they can provide high quality services.

It is recommended that further studies could enlarge the scope of the research by covering more public hospitals from all parts of Greece. In order to have a better idea about the validity of the questionnaire, additional questions measuring customer satisfaction could be included in it. In addition, the development of comparison studies on internal marketing between public and private hospitals should be encouraged.

## Competing interests

The authors declare that they have no competing interests.

## Authors' contributions

EI and CVP developed the original idea for this study. EI extracted the data, performed the statistical analysis and wrote the first draft. CVP provided supervision during the entire process. Both authors made a substantive contribution to writing the final manuscript. All authors have read and approved the final manuscript.

## Author Information

**Efthymios Iliopoulos **is an orthopaedic surgeon trainee in Orthopaedic Department of General Hospital of Veria, Greece. He holds a Medical Diploma of Aristotle University of Thessaloniki, Greece, and an MBA in Health Services Management from Nottingham Trent University (e-mail:prospo81@yahoo.gr).

**Constantinos-Vasilios Priporas **is affiliated with the University of Macedonia, Department of Marketing & Operations Management, Edessa, Greece. He has taught in several Technological Educational Institutes, Colleges and The Greek Open University. He holds a BSBA in marketing from Drexel University (USA), an MBA in marketing and management from Cleveland State University (USA), and a PhD in marketing from University of Newcastle Upon Tyne (UK). He has published in several international academic journals and international conferences. In addition, he is a member of several professional marketing bodies and Country Director of EuroMed Research Business Institute for Greece (e-mail:cpriporas@gmail.com, cpriporas@yahoo.gr).

## Pre-publication history

The pre-publication history for this paper can be accessed here:

http://www.biomedcentral.com/1472-6963/11/261/prepub

## Supplementary Material

Additional file 1 **Appendix 1**. This additional file contains a copy of the questionnaire used in our study. Click here for file
