# Supplementary material for: The effect of internal marketing on job satisfaction in health services: a pilot study in public hospitals in Northern Greece
Source: BMC Health Serv Res. 2011 Oct 9;11:261. doi: 10.1186/1472-6963-11-261 (PMC3200181; doi:10.1186/1472-6963-11-261)
Supplement: Additional file 1 — Appendix 1. This additional file contains a copy of the questionnaire used in our study. [file 1472-6963-11-261-S1.DOC]

**Appendix 1**

**Measurement scales and Demographics**

**Internal Marketing**

*Foreman and Money (1995) (1=Totally Disagree and 5= Totally Agree)*

1. Our hospital offers employees a vision that they can believe in
2. The administration communicates our hospital’s vision well to employees
3. This hospital prepares the employees to perform well
4. Our hospital views the development of knowledge and skills in employees as an investment rather than a cost
5. Skill and knowledge development of employees happens as an ongoing process in our hospital
6. This hospital teaches employees “why they should do things” and not just “how they should do things”
7. In our hospital we go beyond training and educate employees as well
8. The performance measurement and reward systems encourage employees to work together
9. The administration measures and rewards employee performance that contributes most to our hospital’s vision
10. The administration uses the data gathered from employees to improve their jobs, and to develop the strategy of the hospital
11. Our hospital communicates to employees the importance of their service roles
12. In our hospital, those employees who provide excellent service are rewarded for their efforts
13. In this hospital, the employees are properly trained to perform their services roles
14. This hospital has the flexibility to accommodate the differing needs of employees
15. In this hospital is placed considerable emphasis on communicating with employees

**Job satisfaction**

Stamps and Piedmonte (1986) *(1=Totally Disagree and 5= Totally Agree)*

1. My present pay is satisfactory
2. I think that an increase at my salary is needed
3. Considering what is expected of me where I work, the pay I get is reasonable
4. Excluding myself, it is my impression that a lot of my coworkers are dissatisfied with their pay
5. Compared with other hospitals, where I work our pay is fair
6. There is a good deal of teamwork and cooperation on my job
7. There is a lot of conflict in relationship between colleagues
8. Most people appreciate the importance of my work
9. I think I could do a better job if I didn’t have so much to do all the time
10. There is too much paper work required of me where I work
11. I make most of my own decisions regarding my work
12. I am supervised more closely than necessary
13. Where I work doctors cooperate sufficiently with nurses
14. Doctors where I work generally understand and appreciate what nurses do

**DEMOGRAPHICS**

**Gender**

□ Male □ Female

**Occupation specialty**

□ Physician (Doctor) □ Nurse □ Paramedics

**Job position in the Hospital**

□ Director □ Subordinate

**Your Age ………..**

**Work experience (in years)………**

**Work experience in this hospital (in years)………**

**Type of contract**

□ Permanent □ Time defined

**Education**

□ PhD

□ Masters Degree

□ University Degree

□ Τechnological Educational Institute Degree

□ Vocational Degree (2 years Degree)

**Family Status**

□ Married □ Single
